# Brivaracetam Versus Oxcarbazepine in Pediatric Focal Epilepsy: A Comparative Study

**DOI:** 10.7759/cureus.86798

**Published:** 2025-06-26

**Authors:** Divya Sachdeva, Utkarsh Bansal, Pooja Semwal, Prabhat Kumar, Aishwarya Bajpai, Ekansh Rathoria, Nandita Prabhat

**Affiliations:** 1 Pediatrics, Hind Institute of Medical Sciences, Barabanki, IND; 2 Pediatric Gastroenterology, Hind Institute of Medical Sciences, Barabanki, IND; 3 Pediatrics, National Capital Region Institute of Medical Sciences, Meerut, IND; 4 Pediatrics, Hind Institute of Medical Sciences, Sitapur, IND; 5 Neurology, Hind Institute of Medical Sciences, Barabanki, IND

**Keywords:** aed (anti-epileptic drugs), antiseizure medication, childhood epilepsy, focal impaired awareness seizure, focal motor seizures, focal seizure, neurocysticercosis, newer antiepileptic drugs, pediatric epilepsy, seizure-free outcome

## Abstract

Aim and objectives

Focal seizures impact a significant portion of the pediatric population. The efficacy and safety of current antiseizure medications (ASMs) remain subjects of ongoing debate, prompting the evaluation of newer, safer drugs. Brivaracetam (BRV), a novel ASM, has yet to be fully compared to established first-line ASMs, particularly in pediatric patients. Thus, this study was planned to compare the efficacy, safety, and tolerability of oxcarbazepine (OXC) and BRV in children with focal epilepsy.

Materials and methods

Pediatric patients with focal seizures, aged four to 14 years, were prospectively enrolled in this comparative study after informed parental consent. Patients with seizures due to metabolic abnormalities or concomitant chronic systemic illnesses were excluded. A complete history-taking, physical examination, anthropometry, and relevant investigations (including electroencephalogram and neuroimaging) were done in all cases. Participants were randomized to receive either OXC or BRV and followed for up to six months. Participants were followed monthly for compliance, anthropometric measurements, seizure recurrence, and any clinical adverse effects. Seizure-free outcome at six months was the primary outcome. Treatment-emergent adverse events (TEAEs) were recorded and analyzed statistically as secondary outcomes.

Results

Of the 327 participants, 292 completed the study (OXC: 144; BRV: 148). Seizure-free outcome rates at three and six months were 83.3% (120/144) and 65.3% (94/144) in the OXC group, and 77.7% (115/148) and 73.0% (108/148) in the BRV group, respectively, with no statistically significant difference between the groups. Both medications were well tolerated. Somnolence, behavioral abnormalities, and headache were the most common adverse effects. However, the OXC group had a significantly higher incidence of hyponatremia and weight gain compared to the BRV group.

Conclusion

No significant difference in seizure-free outcome rates or overall adverse effects between the groups was seen, indicating that BRV is comparable to OXC in treating pediatric focal epilepsy and can be used in cases where OXC is non-tolerated or has adverse effects.

## Introduction

Epilepsy is one of the most prevalent neurological disorders in childhood, affecting approximately 0.5%-1.0% of the pediatric population [1]. According to the International League Against Epilepsy (ILAE), focal seizures originate in one hemisphere of the brain and involve only a localized area [2]. These seizures can present as either focal awareness or focal impaired awareness seizures. Accurate classification is critical for guiding treatment and determining prognosis. Despite the availability of various antiseizure medications (ASMs), long-term seizure control remains a challenge, with over 25% of children failing to achieve sustained remission [3].

The ongoing search for effective and safer treatments has led to the discovery of newer ASMs. Oxcarbazepine (OXC), a well-established ASM, has demonstrated significant efficacy in treating focal seizures in children, both as monotherapy and as adjunctive therapy [4,5]. Brivaracetam (BRV), a newer ASM and a derivative of levetiracetam, has recently been approved as an alternative treatment for focal epilepsy in both adults and children. Early data suggest that BRV may provide greater seizure reduction compared to placebo [6,7]. However, its efficacy as a monotherapy in pediatric focal epilepsy requires further investigation. There are no studies evaluating its relative efficacy against other alternative established treatments, particularly first-line drugs like OXC. The present study is an attempt to fill this gap in the literature. Therefore, this study was conducted to compare the efficacy, safety, and tolerability of BRV versus OXC monotherapy in treating focal epilepsy in children aged four to 14 years.

## Materials and methods

This institution-based comparative analytical study was conducted in the Department of Pediatrics at a tertiary care center over a 12-month period. The study included children aged four to 14 years who were diagnosed with focal onset epilepsy (either new onset or inadequately treated). Written informed consent was obtained from parents/guardians, and assent was taken from children above seven years. Confidentiality of patient information was maintained throughout the study. Inadequately treated focal epilepsy was defined as epilepsy with a misdiagnosis or inappropriate medication selection. Children having seizures due to metabolic abnormalities or concomitant chronic systemic illnesses were excluded from the study.

The sample size was calculated by considering the efficacy of OXC as 78.12% and assuming a margin of 13% non-inferiority for BRV [8]. For a significance level (α) of 0.05, statistical power (1-β) of 80%, and an allocation ratio of 1:1, the sample size calculated was 118 in each study group using the formula proposed by Fleiss [9].

All patients underwent a comprehensive clinical evaluation, including a detailed history of the seizure semiology, including the type, number of seizure episodes in the last month, age at onset, details of previous ASM, and an anthropometric profile. A general and systemic examination, followed by the relevant investigations, was performed. Children were managed following a standardized treatment protocol for seizures. An electroencephalogram (EEG) and neuroimaging (computed tomography scan/magnetic resonance imaging) were done within 24 hours of stabilization.

**Figure 1:**
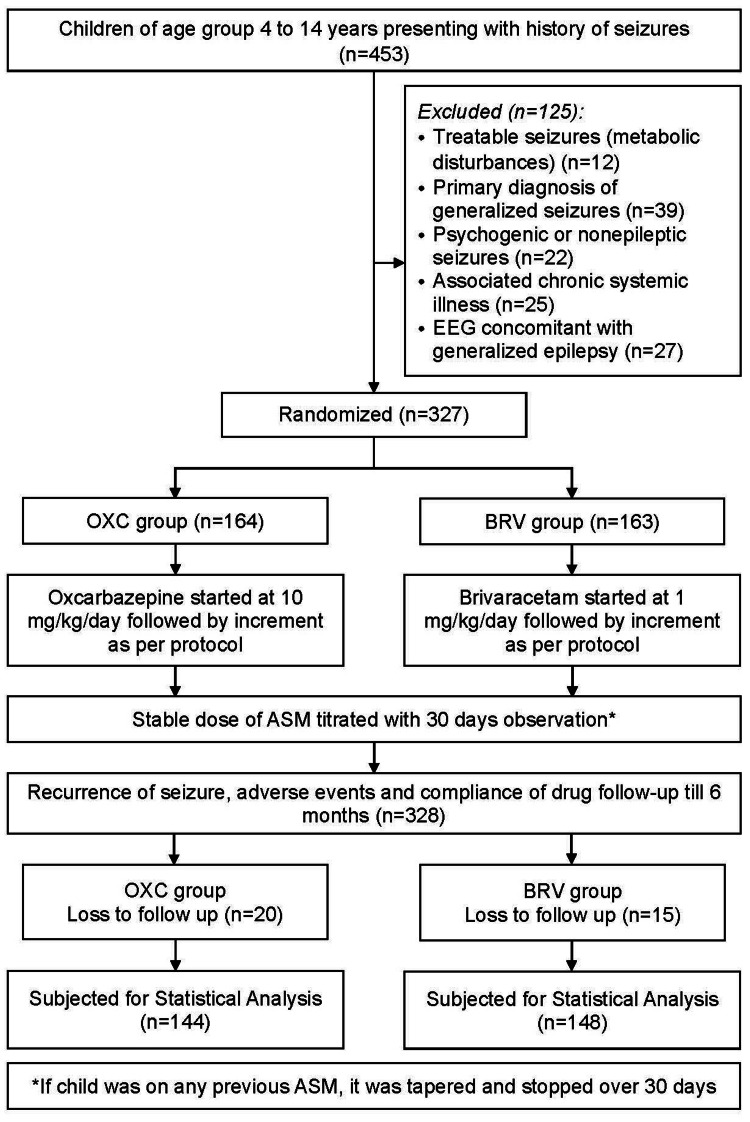
Flow of recruitment of participants in the study OXC: oxcarbazepine, BRV: brivaracetam; EEG: electroencephalogram; ASM: antiseizure medication

In our study, participants were randomly assigned to two groups using a computer-generated randomization table with simple randomization, done by MS Excel version 2010 (Microsoft Corporation, Redmond, WA, United States) by applying the appropriate algorithm. OXC group was initiated on oral OXC, beginning at 10 mg/kg/day, and titrated up by 10 mg/kg/day over one to two weeks to a maximum dosage of 60 mg/kg/day (maximum 1200 mg/day), if needed. The BRV group received BRV, initiated at 1 mg/kg/day, titrated up by 1 mg/kg/day over one to two weeks to a maximum dosage of 5 mg/kg/day (maximum 200 mg/day) if required, as shown in Figure 1. No changes or additions of concomitant ASMs were allowed during the study, and all earlier ASMs (if any) were tapered and stopped over 30 days before starting the study period.

Patients were followed up monthly. At each visit, compliance, anthropometric measurements, seizure recurrence, and any clinical adverse effects were recorded. Parents were instructed to maintain a seizure diary and, if feasible, to video record seizure episodes for precise reporting. Behavioral assessments were conducted at baseline and during follow-up visits using a checklist to monitor potential behavioral changes. Treatment-emergent adverse events (TEAEs), if any, were noted. TEAEs were defined as any adverse event that occurred or worsened in severity after the initiation of the study drug. Specific adverse events, including somnolence, headache, hyponatremia, behavioral abnormalities, and weight gain, were analyzed to assess the safety profile of each drug. Blood tests for sodium levels were done at initiation and three months. The proportion of seizure-free patients was evaluated at three and six-month follow-up intervals. Seizure freedom was defined as the complete absence of clinical seizure episodes during the follow-up period, as documented through patient/caregiver reports and verified by clinical evaluation. The primary outcome of the study was the proportion of patients who achieved seizure freedom at a six-month follow-up period. TEAEs were considered as secondary outcome measures in this study.

Descriptive variables were analyzed using the Statistical Package for the Social Sciences (SPSS) software 22.0 (released 2013; IBM Corp., Armonk, NY, United States). Categorical variables were expressed as percentages (%), while quantitative variables were presented as the mean and standard deviation (SD). For comparing two groups, the independent t-test was used for numerical data and the chi-squared test for categorical data. The Mann-Whitney U test was applied for the non-parametric data. A p-value less than 0.05 was considered statistically significant.

## Results

A total of 327 participants were enrolled and screened according to the inclusion and exclusion criteria. Thirty-five patients were lost to follow-up. A total of 292 children completed the study, consisting of 129 (44.2%) females, with a mean age of 9.50±3.04 years. The majority of patients, 166 (57%), fell within the four to 10 years age group. The baseline characteristics of the study population are presented in Table 1.

**Table 1:** Baseline characteristics of the enrolled participants Data presented as n (%) or^ a^mean (SD) OXC: oxcarbazepine; BRV: brivaracetam; SD: standard deviation; ꭓ^2^: chi-square test value; t: t-test value; U: Mann-Whitney U test value

Characteristics	OXC group (n=144)	BRV group (n=148)	Statistical test value	P-value
Male	87 (60.4)	76 (51.4)	ꭓ^2^=2.079	0.149
Mean age (years)^a^ (range 9-14 years)	10.13 (2.90)	8.89 (3.04)	t=1.771	0.080
Mean age at onset (years)^a^ (range 9-14 years)	9.73 (2.79)	8.64 (3.13)	t=1.563	0.134
BMI				
< -2 SD	84 (58.3)	72 (48.6)	ꭓ^2^=0.717	0.699
Within normal	55 (38.2)	73 (49.3)		
>2 SD	5 (3.4)	3 (2.02)		
Number of seizure episodes (per day)				
1	35 (24.3)	40 (27.0)	U=12	1.0
2	44 (30.5)	49 (33.1)		
3	33 (22.9)	28 (18.9)		
4	24 (16.6)	15 (10.1)		
≥5	8 (5.6)	12 (8.1)		
Previous anti-seizure medication				
None	100 (69.4)	113 (75.7)	ꭓ^2^=2.966	0.397
Valparin	44 (30.6)	27 (18.9)		
Phenytoin	0 (0)	4 (2.7)		
Valparin+phenytoin	0 (0)	4 (2.7)		

The mean age of onset for focal seizures was 9.19±3.13 years. Most participants 272 (93%) experienced fewer than five seizure episodes before the study. A significant proportion of patients 213 (72.9%) had not received prior ASM. Among those who had, sodium valproate was the most commonly used ASM.

An etiological assessment revealed a variety of underlying causes for focal epilepsy in this study. Infectious etiologies were the most prevalent, affecting 241 (82%) children. Neurocysticercosis (NCC) was the most common diagnosis within this category, accounting for 176 (60.3%) cases. Other notable infectious etiologies included tuberculoma 41 (13.7%) and tubercular meningitis 24 (8.2%). Additional etiologies included head trauma (n=15, 5.5%), infarcts (n=4, 1.4%), and unknown etiology (n=33, 11%).

As shown in Table 1, there was no statistically significant difference between the two study groups (OXC vs BRV) in terms of age, sex distribution, anthropometric data, history of ASM use, frequency of seizure episodes, or clinical presentation at the time of enrollment.

The proportion of seizure-free patients at three months was 120/144 (83.3%) in the OXC group and 115/148 (77.7%) in the BRV group. The primary outcome of the study was seizure freedom at six months. The corresponding rates were 94/144 (65.3%) in the OXC group and 108/148 (73.0%) in the BRV group. There was no statistically significant difference between the two groups in terms of seizure-free outcomes at either time point (Table 2). The pre-specified non-inferiority margin was 13%. The difference in the seizure freedom between the two groups was 7.7% (95% CI: -17.8 to 2.4), with the upper limit of the confidence interval remaining below the non-inferiority margin, thereby supporting the non-inferiority of BRV to OXC. The mean dose required for seizure cessation was 17.67±7.29 mg/kg for the OXC group and 1.42±0.41 mg/kg for the BRV group at 6-month follow-up. The median time to seizure-free period was two days in the OXC group and 1.5 days in the BRV group, with no significant difference between the two groups.

**Table 2:** Proportion of patients remaining seizure-free at three and six months Data presented as n (%) OXC: oxcarbazepine; BRV: brivaracetam

Follow-up interval	OXC group (n=144)	BRV group (n=148)	Total (n=292)	ꭓ^2^ Value	P-value
3 months	120 (83.3)	115 (77.7)	235 (80.4)	1.136	0.286
6 months	94 (65.3)	108 (73.0)	200 (68.5)	1.682	0.195

Treatment-emergent adverse events (TEAEs) were observed in 64/144 (47.2%) patients of the OXC group and 68/148 (45.9%) patients of the BRV group, with no statistically significant difference between the groups (Table 3).

**Table 3:** Comparison of TEAEs between the two study groups Bold indicates a statistically significant difference at p≤0.05. Data presented as n (%) OXC: oxcarbazepine, BRV: brivaracetam; TEAE: treatment-emergent adverse event

Adverse effect	OXC group (n=144)	BRV group (n=148)	ꭓ^2 ^Value	P-value
No adverse effect	76 (52.8)	80 (54.1)	0.010	0.91
Somnolence	53 (36.8)	64 (43.2)	1.006	0.32
Headache	20 (13.9)	15 (10.1)	0.651	0.420
Hyponatremia	20 (13.9)	0 (0)	19.944	<0.001
Behavioral abnormalities	19 (13.1)	32 (21.6)	3.035	0.081
Aggression	12 (8.3)	12 (8.1)	0.001	0.97
Weight gain	16 (11.1)	0 (0)	15.319	<0.001
Nausea	8 (5.6)	5 (3.4)	0.382	0.537
Rashes	8 (5.6)	4 (2.7)	0.870	0.351

In the OXC group, the most common adverse event was somnolence, occurring in 53/144 (36.8%) patients, followed by headache, hyponatremia, and behavioral abnormalities. In the BRV group, somnolence was also the most common adverse event, occurring in 64/148 (43.2%) patients, followed by behavioral abnormalities. Patients in the OXC group had a significantly higher incidence of hyponatremia and weight gain compared to those in the BRV group. Importantly, there were no treatment withdrawals due to adverse events in either group, demonstrating 100% treatment tolerability for both OXC and BRV.

## Discussion

ASMs play an important role in the management of focal epilepsy. As per a recent study from India, the point prevalence of epilepsy in children has been reported as 3.53 per 1000 children, with almost 53% having focal epilepsy [10]. At the same time, 34.5% of the first seizures can be of a focal onset [11]. Although several medications have become available in the last decades, the prevalence of refractory epilepsy remains high, and around one-third of patients continue to have incomplete seizure control, prompting the use of newer drugs. This study highlights important findings on the use of BRV in children with focal epilepsy.

The inclusion criteria for our study focused on children aged four to 14 years, as BRV had not yet been clinically evaluated in India for younger age groups. This determined the age profile of our patients. Regarding the sex distribution, a higher incidence of epilepsy in males could explain the predominance of male participants. There was no significant difference between the two study groups for age, sex, anthropometry, anti-seizure medication history, age of onset, frequency of seizure episodes, and clinical presentation at the time of enrolment. This could be owing to strict inclusion criteria and detailed exclusion criteria that helped us to get a relatively homogenous group of patients.

Most children in our study experienced less than five seizure episodes in the last 28 days before enrollment, with a mean age of onset of 9.19±3.13 years. A majority (72.6%) of the participants were treatment-naïve. Sodium valproate, either alone or in combination with phenytoin, was used by 26.1% of the children. In contrast, Visa-Reñé et al. reported that 43.5% of patients had a seizure frequency of more than one episode per day, while Yi et al. noted a seizure frequency of more than 10 episodes in their cohort [12,13]. Compared to these studies, our patient population had a significantly lower seizure frequency. Consequently, our primary treatment goal was to maintain seizure-free status, rather than simply focusing on reducing seizure frequency, which was the aim of many other studies [14-18].

At three months, 77.7% (115/148) in the BRV group and 83.3% (120/144) of patients in the OXC group remained seizure-free, with no significant difference between the two groups. At six months, the seizure-free rates were 73.0% (108/148) in the BRV group and 65.3% (94/144) in the OXC group, again showing no statistically significant difference. Previous studies on OXC have reported wide variability in seizure-free rates, depending on patient profiles and follow-up durations. Hur et al. reported a seizure-free rate of 56.2% at one year, compared to 63.9% at six months in our study; this difference is likely due to the shorter follow-up period [19]. Other studies have reported seizure-free rates ranging from 56% to 91%, depending on the inclusion criteria and patient characteristics [8,13,20]. Despite these variations, a consistent trend of successful outcomes in 60%-80% of cases aligns with our findings. Similar to these studies, our research supports OXC's efficacy in maintaining seizure-free status in children across varying patient profiles and disease characteristics. In our cohort, a high prevalence of NCC (60.3%) and a lower burden of structural lesions might have contributed to better seizure control.

BRV, on the other hand, has limited literature available and exhibits greater variability in treatment response. Studies by Visa-Reñé et al., Ferragut Ferretjans et al., and Schubert-Bast et al. have reported seizure-free rates ranging from 9% to 37.5% [12,15,21]. In contrast, our study demonstrated a higher seizure-free rate of 73% at six months. A relatively higher seizure-free outcome in the present study could primarily be attributed to the dominance of those with non-structural etiologies, for whom additional routine treatment was also being delivered. It may also be due to differences in patient populations, epilepsy characteristics, and healthcare factors, along with strict patient selection, adherence to protocols, and frequent follow-ups.

In terms of adverse effects, both OXC and BRV demonstrated comparable safety profiles. TEAEs were reported in 47.2% of OXC patients and 45.9% of BRV patients. Studies on OXC have generally shown high tolerability. For instance, Hur et al. reported no intolerable adverse events in OXC-treated patients, even with longer follow-up periods [19]. In other studies, adverse event rates have ranged from 9.6% to 30.4% [13,20]. However, one study reported adverse events in as many as 81.9% of OXC patients, with 5.6% experiencing severe effects that led to withdrawal [22]. In contrast, all adverse effects in our study were mild, transient, and well-tolerated, without the need for drug withdrawal. This aligns with the findings of the most recent studies.

In our study, the adverse event rate for BRV was comparable to that of OXC (45.9% vs. 47.2%), consistent with the findings of Visa-Reñé et al. [12]. However, unlike their study, in which 34.7% of patients experienced severe adverse effects leading to treatment withdrawal, none of our patients required discontinuation. Farkas et al. also supported the tolerability of BRV at doses up to 200 mg/day, reporting no significant treatment-related adverse effects [23].

The literature presents a wide range of variability in BRV’s adverse event profile. Few studies report low adverse event rates (12% to 25%), while others, like Liu et al., Lagae et al., and Patel et al., report significantly higher rates (75% to 90%) [14-18,21,23,24]. Although BRV was developed with a focus on both safety and efficacy, this variability across studies indicates the need for more comprehensive data to accurately assess its safety profile.

In terms of specific adverse effects, we observed a higher incidence of hyponatremia and weight gain in the OXC group, which are known side effects of the drug as per the reported studies [20,22]. These adverse effects carry important clinical implications. Hyponatremia may necessitate routine serum sodium monitoring and potential dose adjustments, particularly in younger children or those with concurrent use of other medications affecting electrolyte balance. Weight gain, especially in older children and adolescents, can negatively impact self-esteem and adherence to long-term therapy, influencing overall treatment success. In contrast, BRV was not associated with hyponatremia or weight gain but showed a higher incidence of behavioral issues, such as irritability or aggression, which, although typically mild, warrant close neuropsychiatric monitoring. This is particularly relevant for children with pre-existing behavioral concerns or neurodevelopmental comorbidities.

These differences underline the importance of individualized treatment planning in pediatric epilepsy. For children at risk for metabolic disturbances or electrolyte imbalance, BRV may be a preferable first-line option. Conversely, in children with significant behavioral or psychiatric vulnerability, OXC may be better tolerated. Thus, while both drugs demonstrated comparable efficacy, the tolerability profiles suggest that tailoring therapy based on patient-specific risk factors and comorbidities can optimize outcomes. Balancing seizure control with adverse effects remains central to personalized treatment decisions in pediatric focal epilepsy.

Strengths include a homogeneous patient population, a clear treatment focus, and comprehensive adverse event reporting. However, limitations such as a short follow-up duration, small sample size, and single-center design limit the statistical power and generalizability of the findings to broader populations. Furthermore, as the study was conducted in a tertiary care center in North India, local epidemiological patterns, healthcare-seeking behaviors, and drug availability may not reflect broader or global settings. These methodological constraints may influence both the interpretation and the future applicability of the results.

## Conclusions

In conclusion, no significant differences in seizure-free outcomes or overall TEAEs were observed between BRV and OXC, and both drugs may be used safely for the treatment of pediatric focal epilepsy as a first-line therapy. Notably, BRV was associated with a lower risk of hyponatremia and weight gain, two clinically relevant side effects that often impact long-term adherence and quality of life, especially in the pediatric population. These findings suggest that BRV may be a preferable option in children who are at risk for metabolic disturbances or electrolyte imbalance, while OXC may be preferred in those who have considerable behavioral or mental health risks. However, there is a need for larger, long-term studies to further validate these observations and guide personalized treatment choices in pediatric focal epilepsy.
